# Time Period of Treatment’s Effect on the Association Between Race and Survival in Patients With Malignant Colorectal Adenocarcinoma

**DOI:** 10.7759/cureus.45641

**Published:** 2023-09-20

**Authors:** Juan C Ramirez, Juan C Alvarez, Phillip Cifuentes, Grettel Castro, Noel C Barengo

**Affiliations:** 1 Department of Translational Medicine, Herbert Wertheim College of Medicine, Florida International University, Miami, USA; 2 Department of Medical and Population Health Sciences Research, Herbert Wertheim College of Medicine, Florida International University, Miami, USA; 3 Department of Medicine, Riga Stradins University, Riga, LVA; 4 Department of Global Health, Robert Stempel College of Public Health and Social Work, Florida International University, Miami, USA

**Keywords:** cancer epidemiology, survival rates, standard of care, seer database, racial and ethnic disparities, colorectal cancer

## Abstract

Background: Colorectal cancer is one of the most common malignancies diagnosed in the United States, with 126,240 new cases diagnosed in 2020. Past studies have shown that disparities may exist between certain patient populations, but it is unknown how they are affected over time as treatments evolve. The purpose of this study was to determine whether the decade of treatment modifies the association between race and five-year survival in adults diagnosed and treated for malignant colorectal adenocarcinomas since the 1970s.

Methods: This was a non-concurrent retrospective cohort study using data from the Surveillance, Epidemiology, and End Results (SEER) database of the National Cancer Institute. The inclusion criteria involved patients with primary malignant colorectal adenocarcinoma between the years 1975 and 2018. Exclusion criteria included previous malignancies or missing information on any of the variables. The exposure variable was the patient’s race, and the main outcome variable was average five-year survival rates. The effect modifier was the time period in which the patient received treatment. The covariates of the study included age, sex, Hispanic status, surgical intervention recommendation, and disease stage. Unadjusted and adjusted hazard ratios (HRs) and corresponding 95% confidence intervals (CIs) were calculated using Cox regression models.

Results: As the interaction term between race/ethnicity and year of diagnosis was statistically significant, the data were stratified according to year of diagnosis. Black patients in both time periods had a higher mortality rate from malignant colorectal carcinoma after adjustment for the covariates (1975-1990: HR 1.10, 95% CI 1.06-1.15; 1991-2018: HR 1.19, 95% CI 1.16-1.23) when compared with White patients. American Indian, Alaskan Native, and Asian patients were found to have lower mortality in both time periods when compared with White patients (1975-1990: HR 0.90, 95% CI 0.85-0.95; 1991-2018: HR 0.93, 95% CI 0.89-0.96).

Conclusion: Our data found that despite the evolution in the standard of care treatment for malignant colorectal adenocarcinoma since the year 1975, Black patients had lower five-year survival rates when compared with their White counterparts as well as increased rates of being diagnosed with this disease. Overall, addressing these disparities in colorectal cancer outcomes is critical for improving public health and reducing healthcare costs.

## Introduction

Colorectal cancer is one of the most common malignancies diagnosed in both males and females in the United States. [1]. This form of cancer can be extremely aggressive and usually only becomes symptomatic in the later stages of the disease. Colorectal cancer ranks as the fourth most deadly cancer worldwide, with nearly 900,000 deaths reported annually, in large part due to the symptomatology presenting when the disease is in the later, more aggressive stages [2]. The median age for diagnosis in 2018 was 68 years old, and it is associated with several etiologies, including heritable and non-heritable reasons [3]. About 5-15% of all colorectal cancers can be attributed to inheritable diseases, including hereditary non-polyposis colorectal cancer, familial adenomatous polyposis, MUTYH-associated polyposis, and Peutz-Jeghers syndrome [4]. The other 75-85% of colorectal cancers are sporadic and have no hereditary component to their etiology. Non-hereditary forms of colorectal cancer are usually induced by DNA mutations in the cells that form the colonic mucosa, causing cellular overproliferation and eventually the formation of small neoplasms called polyps, which can be present in most cases [5]. Although many of these mutations in colonic mucosa DNA are considered idiopathic, there are several modifiable risk factors that play a role in inducing disease. Some of these risk factors are common in Western civilization, such as smoking tobacco, overeating/obesity, increased levels of inactivity, diets poor in nutrition, and excessive ingestion of alcohol [6]. Depending on the stage of colorectal cancer, it can be treated with several treatment options. Surgical interventions such as a partial colectomy are used for early-stage colorectal cancers (Stages I-III). Adjuvant chemotherapy regimens are utilized for Stage II and above states. Additional interventions, such as lymph node (LN) resections or resections of distal sites of metastases, are saved for higher-stage diseases such as Stage III and above [7]. 

According to the National Cancer Institute (NCI)’s Surveillance, Epidemiology and End Results (SEER) program, from 2000 to 2018, there were an estimated 225,566 patients, aged 18-85, identifying as either White or Black, with a diagnosis of primary malignancy of colorectal adenocarcinoma. Of these, 89.7% identified as White and 10.3% identified as Black. From that time, 90,881 deaths were observed, with 80,486 deaths among White patients with a median survival time of 48 months. Among Black patients, 10,395 deaths were observed with a median survival time of 35 months. Overall, there appears to be a discrepancy in the median survival time between Whites and Blacks of approximately 13 months. Studies show that incidence rates of colorectal carcinoma (CRC) are highest in Black populations when compared to other races [3]. It is important to understand why these trends exist to better guide public preventative measures and colorectal cancer awareness. 

The goal of this study is to analyze the databases to potentially find specific trends such as colorectal cancer five-year survival rates of the Black population when compared to other ethnicities. This information would help guide public health policy in the distribution of resources, such as screenings and preventive care knowledge, to at-risk populations.

## Materials and methods

Study design and population

This was a retrospective cohort study consisting of a secondary analysis of the SEER database. SEER is a program of the National Cancer Institute focused on the surveillance of cancer and other malignancies in the United States. The data on mortality reported by SEER are provided through a collaborative arrangement between the National Center for Health Statistics (NCHS) and the U.S. Census Bureau for the demographics of the population [8]. The NCHS also acquires self-reported data using questionnaires. The SEER program provides a limited-use data set (formerly called the public use data file) for additional analyses by researchers and the public [9].

Since the early 1970s, SEER has collected cancer incidence data from population-based cancer registries covering approximately 47.9% of the U.S. population [8]. All patients with a diagnosis of malignant colon adenocarcinoma recorded in the SEER database between the years 1975 and 2018 that met the study’s inclusion criteria were included in the study. The inclusion criteria were patients with a diagnosis of malignant colonic adenocarcinoma (CD-10-C18.6) aged 18-85 and whose diagnosis of colonic adenocarcinoma was a primary tumor/malignancy between 1975 and 2018. Excluded from this study were those with previous malignancies of the gastrointestinal tract or any part of the body that is not the colon or rectum, as well as any patients who were missing information on any of the variables used in this study. The final study population consisted of 215,884 patients from an initial collection of 219,642.

Variables

The main exposure variable of this study was the race of the patient. Patients were categorized as White, Black, or Other, which included a combination of patients who were identified as Asian, Alaskan (AK) Native, or Native American in the SEER database. The main response variable was the average cancer-specific five-year survival rate since the time of diagnosis, and this outcome was measured in months. The effect modifier will be the decade in which the patient received treatment. Starting from the year 1991, laparoscopic surgery was incorporated into the guidelines for colorectal cancer treatment [10]. As a result, this has been designated as the cutoff period for the effect modifier’s decade of diagnosis, given that survival outcomes may be expected to be altered by this intervention. The effect modifier of the decade of diagnosis was divided into two time periods: 1975-1990 and 1991-2018. Covariates of the study were age, biological sex, Hispanic ethnicity, the presence or absence of a recommendation for surgical intervention, and the stage of disease represented by the degree of LN involvement. The age of the patients was broken down into three groups: those 49 years and younger, those between the ages of 50 and 64, and those 65 and older. Biological sex was dichotomized into male and female groups. Hispanic ethnicity was categorized based on whether the patient identified as Hispanic or not. Surgical treatment was categorized into three groups: patients who received the recommended surgical intervention, those who did not receive the recommended surgical intervention, and those for whom surgical intervention was not recommended for various reasons. The stage of disease denoted by LN involvement was categorized into whether there was local, regional, or distant LN involvement.

Statistical analysis

STATA software (StataCorp LLC, College Station, TX, United States) was used to perform the statistical analyses of this study. Percentages were used to analyze the nominal variables. A descriptive analysis was initially conducted to check for missing information or variables and for the distributions of categorical variables. A bivariate analysis was conducted to examine the association of potential confounders with the main independent variable, patient race. Log-rank tests were performed to assess the differences in the survival distribution for the independent variable. Kaplan-Meier curves were generated for the survival distribution of the sample by race between the two time-of-diagnosis groups, 1975-1990 and 1991-2018, respectively, as well as one curve for all-time survival. Collinearity diagnostics were performed as well prior to conducting multivariable analysis. Cox regression was performed to calculate the unadjusted and adjusted hazard ratios (HRs) with corresponding 95% confidence intervals (CIs). The proportional hazard assumptions were assessed graphically as well. The interaction was tested by adding an interaction term, race*time of diagnosis, to the statistical model.

Ethical aspects

There are minimal ethical aspects to this project due to the data being extracted from the NCI's SEER database, for which only statistical information was collected. The SEER database organizes its data without any components or forms of protected health information, and all forms of identifiable information are removed. Our data did not include any sort of information that could have been used to identify patients; therefore, a review and approval by an IRB committee were unnecessary.

## Results

**Figure 1:**
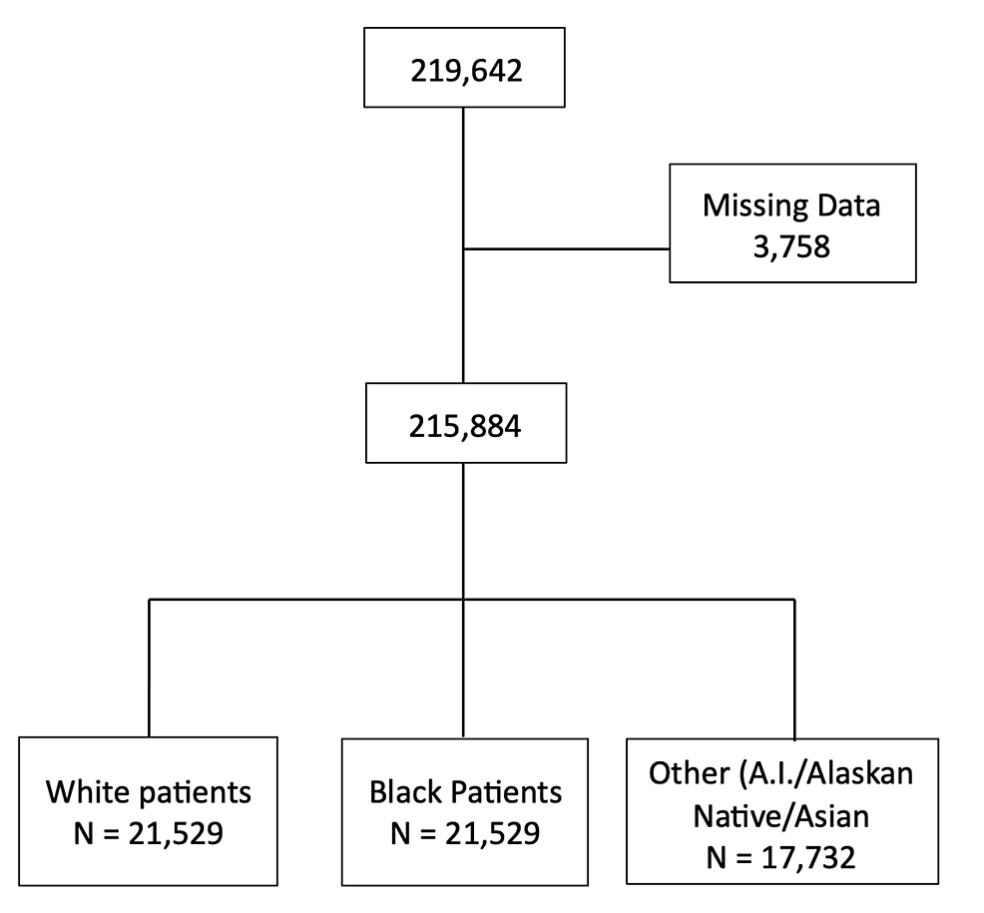
Sample of the study. AI: American Indian (Native American); AK: Alaskan.

The total number of potential participants of malignant CRC patients in the SEER database between 1975 and 2018 was 219,642 (Figure 1). A total of 215,884 were left for the analysis after applying the exclusion criteria.

Table 1 shows the distribution of baseline characteristics of adult malignant CRC patients in the SEER database from 1975 to 2018 according to race. All baseline characteristics of the study participants (age, sex, Hispanic ethnicity, surgical intervention recommendation, staging of disease based on degree of LN involvement, and time of diagnosis) were differently distributed according to race (p<0.001).

**Table 1:** Baseline characteristics of the sample of malignant CRC patients in the SEER database (1975-2018). CRC: colorectal carcinoma; SEER: Surveillance, Epidemiology and End Results; AI: American Indian; AK: Alaskan; LN: lymph node.

	Race			
	White	Black	Other (AI/AK Native/Asian)	p-value
	(n=176,623)	(n=21,529)	(n=17,732)	
	%	%	%	
Age (years)				<0.001
≤49	7.96	13.44	12.2	
50-64	28.41	36.95	33.23	
65-84	63.63	49.61	54.57	
Sex				<0.001
Female	48.8	52.57	46.26	
Male	51.2	47.43	53.74	
Ethnicity				<0.001
Non-Hispanic	94.54	99.16	98.55	
Hispanic	5.46	0.84	1.45	
Surgical intervention				<0.001
Surgery performed	91.79	85.68	91.24	
Surgery not recommended	4.81	9.41	6.32	
Surgery recommended, not performed	3.4	4.91	2.44	
Stage of disease based on LN involvement				<0.001
Localized	31.64	26.67	31.5	
Regional	44.89	42.29	46.25	
Distant	23.47	31.04	22.25	
Time period of diagnosis				0.001
1975-1990	41.42	29.44	22.99	
1991-2018	58.58	70.56	77.01	

The highest percentage of participants were older than 65 years, independent of race (White: 63.63%, Black: 49.61%, Other (Asian, AK Native, or AI): 54.57%). In regard to biological sex, there was a statistically significant difference among the races with White males at 51.2% versus White females at 49.7%, (p<0.001), Black females at 52.57% versus Black males at 47.43% (p<0.001), and Other males at 53.74% versus Other females at 46.26% (p<0.001). Most patients in the study were non-Hispanic (White: 94.54%, Black: 99.16%, Other: 98.55%, p<0.001). A vast majority of patients received a recommendation for surgical intervention to be performed (White: 91.79%, Black: 85.68%, Other: 91.24%, p<0.001). However, Black patients had a statistically significant higher prevalence of not receiving a recommendation for surgical intervention than White patients or those of other races (Black: 9.41%, White: 4.81%, Other: 6.32%, p<0.001). There was a statistically significant difference in the distribution of disease stage by LN involvement according to race. The percentage of adenocarcinomas with regional LN involvement was 45% (White patients), 42% (Black patients), and 46% (patients of other races; p<0.001), respectively. Black patients (31%) had a statistically significantly higher percentage of distant LN involvement compared with other races (White: 23.47%, Other: 22.25%, p<0.001). Most CRC diagnoses included in our study were made between the years 1991 and 2018 across all race groups (White: 58.58%, Black: 70.56%, Other: 77.01%, p<0.001).

Table 2 shows the percentiles of the survival time in months according to race. The 25th percentile showed that 75% of White patients were alive at 24 months (95% CI: 24-24), which was six months longer when compared with Black patients at 18 months (95% CI: 17-18). After seven months of follow-up, 90% of White patients were alive (95% CI: 7-7). Also, 90% of Black patients were alive after five months (95% CI: 5-5). Patients of other race groups consistently had a large portion of their population survive for more months when compared with both White and Black patients, with 75% of patients alive at 31 months (95% CI: 30-33) and 90% of patients alive after 10 months (95% CI: 10-11). The p-value of the log-rank test showed that survival differed statistically significantly according to race (Table 3).

**Table 2:** Median survival time percentiles (in months) by race. CI: confidence interval; AI: American Indian (Native American); AK: Alaskan.

Race	No. of subjects	25% (95% CI)	20% (95% CI)	15% (95% CI)	10% (95% CI)
White	168,850	24 (24, 24)	17 (17, 17)	12 (11, 12)	7 (7, 7)
Black	20,354	18 (17, 18)	13 (13, 14)	9 (9,9)	5 (5, 5)
Other (AI/AK Native/Asian)	17,154	31 (30, 33)	22 (21, 23)	16 (15, 17)	10 (9, 10)

**Table 3:** Log-rank test for equality of survival function. AI: American Indian (Native American); AK: Alaskan; LN: lymph node.

Characteristics	Events observed		Events expected	Log-rank X^2^ test	p-value
Race				606.36	<0.001
White		60,244	61033.50		
Black		8498	6759.79		
Other (AI/AK Native/Asian)		5392	6330.70		
Total		74,124	74,124		
Age (years)				98.96	<0.001
≤49		6646	6971.32		
50-64		22,143	23139.81		
65-84		45,335	44012.86		
Total		74,124	74124		
Sex				45.17	< 0.001
Male		38,364	37454.27		
Female		35,760	36669.73		
Total		74,124	74124		
Hispanic ethnicity				6.73	0.0095
Yes		70,854	70706.73		
No		3270	3417.27		
Total		74,124	74124		
Surgical intervention				40,044.79	< 0.001
Surgery performed		61,373	70934.33		
Surgery not recommended		7567	1450.47		
Surgery is recommended, but not performed		4436	991.2		
Total		74,124	74124		
Disease stage (LN involvement)				109,788.95	< 0.001
Localized		6639	26693.87		
Regional		26,873	34642.18		
Distant		36,607	8782.95		
Total		70,119	70119		
Year of diagnosis				1550.24	< 0.001
1975-1990		33,709	28522.68		
1991-2018		40,415	45601.32		
Total		74,124	74124		

Black patients in both time periods had higher mortality from malignant CRC after adjustment for the covariates (1975-1990: HR 1.10, 95% CI: 1.06-1.15; 1991-2018: HR 1.19, 95% CI: 1.16-1.23) when compared with White patients (Table 4). American Indian (AI), AK Native, and Asian patients were found to have lower mortality rates when compared with White patients. The data revealed a 10% decrease in mortality for those diagnosed between 1975 and 1990 (HR 0.90, 95% CI 0.85-0.95) and a 7% decrease between 1991 and 2018 (HR 0.93, 95% CI 0.89-0.96). There was a statistically significant decrease in mortality in Hispanic patients in the later time of diagnosis group (1991-2018: HR 0.93, 95% CI: 0.89-0.97). There was no statistically significant association between sex and survival after adjusting for covariates. As expected, there was an increased mortality in patients who were recommended not to receive surgical intervention reason and in those who did not receive surgical intervention, despite current recommendations. However, mortality slightly decreased over time for those who were not recommended to have surgery (1975-1990: HR 2.56, 95% CI 2.32-2.82; 1991-2018: HR 2.43, 2.36-2.51). For patients who were recommended to have surgery but did not proceed with it, the mortality increased (1975-1990: HR 18.00, 95% CI 17.31-18.72; 1991-2018: HR 18.48, 95% CI 17.78-19.20) with respect to time. 

**Table 4:** Comparison of the unadjusted and adjusted HRs of the variables stratified by the time of diagnosis. AI: American Indian (Native American); AK: Alaskan; HR: hazard ratio; CI: confidence interval; LN: lymph node. ^a^Reference group. ^b^Surgical intervention performed as a treatment for malignancy. *Statistically significant at p<0.05.

	Time of diagnosis			
Characteristics	1975-1990		1991-2018	
	Unadjusted	Adjusted	Unadjusted	Adjusted
	HR (95% CI)	HR (95% CI)	HR (95% CI)	HR (95% CI)
Race				
White	Ref.^a^	Ref.	Ref.	Ref.
Black	1.25 (1.20-1.29)	1.10 (1.06-1.15)*	1.37 (1.33-1.41)	1.19 (1.16-1.23)*
Other (AI/AK Native/Asian)	0.88 (0.84-0.93)	0.90 (0.85-0.95)*	0.92 (0.89-0.95)	0.93 (0.89-0.96)*
Age (years)				
65-84	Ref.	Ref.	Ref.	Ref.
≤49	0.96 (0.92-1.00)	0.81 (0.77-0.85)*	0.96 (0.93-0.99)	0.64 (0.62-0.67)*
50-64	0.96 (0.93-0.98)	0.86 (0.84-0.89)*	0.92 (0.90-0.94)	0.71 (0.70-0.73)*
Sex				
Male	Ref.	Ref.	Ref.	Ref.
Female	0.95 (0.93-0.97)	0.97 (0.95-1.00)	0.95 (0.93-0.97)	0.99 (0.97-1.01)
Ethnicity				
Non-Hispanic	Ref.	Ref.	Ref.	Ref.
Hispanic	1.01 (0.95-1.09)	1.06 (0.98-1.13)	1.00 (0.97-1.05)	0.93 (0.89-0.97)*
Surgical intervention^b^				
Surgery performed	Ref.	Ref.	Ref.	Ref.
Surgery not recommended	7.48 (6.82-8.21)	2.56 (2.32-2.82)*	7.54 (7.35-7.75)	2.43 (2.36-2.51)*
Surgery recommended, not performed	6.18 (5.94-6.43)	3.04 (2.90-3.17)*	4.25 (4.04-4.47)	2.95 (2.78-3.12)*
Staging of disease based on LN involvement				
Local	Ref.	Ref.	Ref.	Ref.
Regional	3.25 (3.12-3.38)	3.31 (3.19-3.44)*	3.10 (2.98-3.22)	3.24 (3.12-3.36)*
Distant	19.66 (18.91-20.42)	18.00 (17.31-18.72)*	20.36 (19.62-21.13)	18.48 (17.78-19.20)*

**Figure 2:**
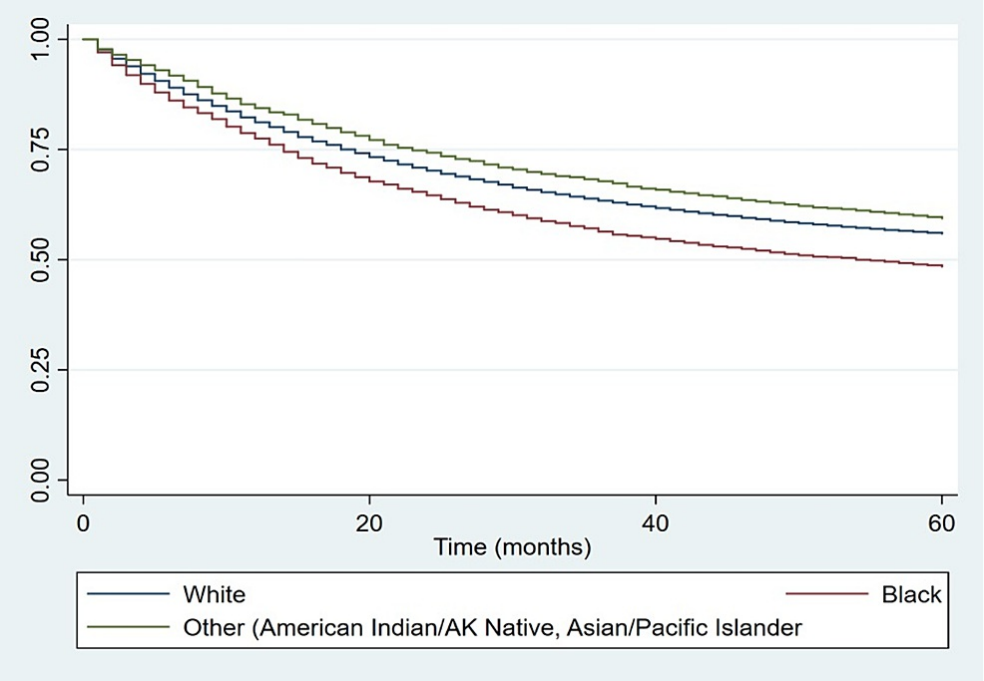
Kaplan-Meier curve for patients diagnosed with CRC by race, 1975-1990. CRC: colorectal carcinoma; AK: Alaskan.

**Figure 3:**
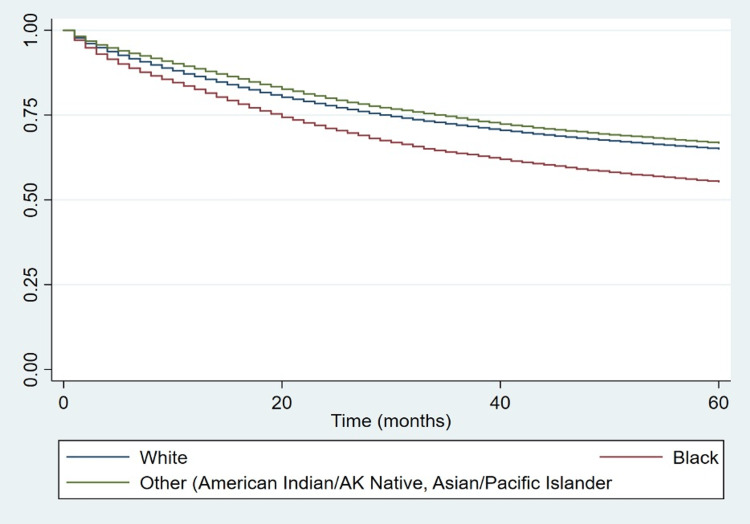
Kaplan-Meier curve for patients diagnosed with CRC by race, 1991-2018. CRC: colorectal carcinoma; AK: Alaskan.

**Figure 4:**
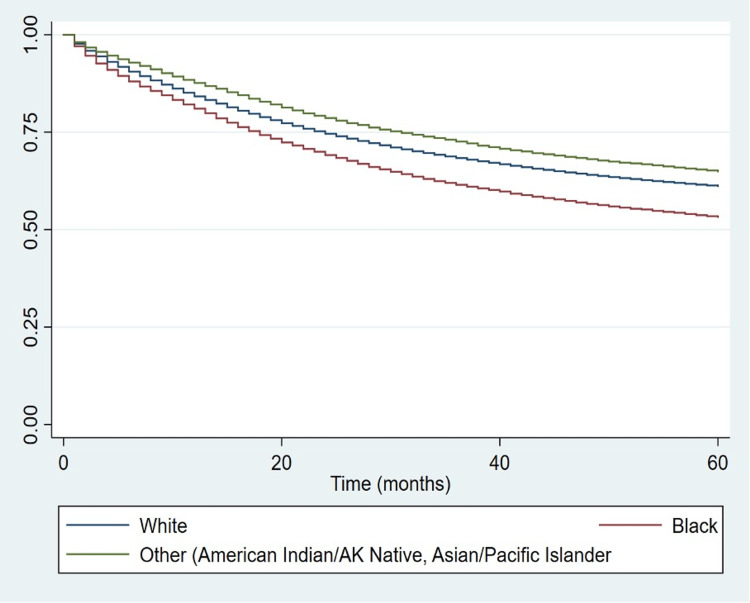
Kaplan-Meier curve for patients diagnosed with CRC by race (all time). CRC: colorectal carcinoma; AK: Alaskan.

Figures 2, 3 display the Kaplan-Meier survival curve of malignant CRC patients for the periods of 1975-1990 and 1991-2018, respectively. Figure 4 shows the Kaplan-Meier survival curve of malignant CRC patients throughout the entire time period of 1975-2018. The log-rank test showed that the difference in the survival of the patients based on race was statistically significant (Table 3). All three curves revealed that Black patients had a shorter median survival time whether at the 50th percentile or at any other lower percentile compared with White patients and those of other races.

The interaction term between race/ethnicity and year of diagnosis was statistically significant, and the data were stratified according to year of diagnosis.

## Discussion

Our data found that, despite the evolution in the standard of care treatment for malignant colorectal adenocarcinoma since the year 1975, Black patients had lower five-year survival rates when compared with their White counterparts as well as had increased rates of being diagnosed with this disease. Asians, AIs, and AK Natives were found to have reduced mortality compared with White patients. It was also noted that Hispanic ethnicity was associated with decreased mortality in recent years when adjusted for covariates. Our data found no statistically significant association between sex and survival from CRC after adjusting for covariates with respect to the time of diagnosis. Also, as expected, the data revealed that mortality increased in those patients who did not receive surgical intervention, whether it was recommended or not. However, mortality with respect to the time of diagnosis did decrease minimally. It was also noted that the advanced stage of disease at the time of diagnosis with respect to LN involvement was associated with increased mortality.

Our results are consistent with previous studies that examined the differences in mortality rates between patients of different races suffering from CRC. One study that examined the impact of income and social mobility on CRC outcomes and treatment found that Black patients had a 21% increased risk of presenting with an advanced stage of cancer when compared to White patients [6]. That study controlled for age and biological sex, such as our study, but went further in controlling income mobility between the races of the patients [6]. Another study exhibited higher rates of late-stage CRC diagnosis when compared to White patients, with an 18% higher chance [11]. One study performed in 2021 reported that despite higher rates of guideline-concordant care for CRC, Black patients experience a higher risk of recurrence and mortality from the disease compared to White patients [12]. This distinct difference in mortality rate is concurrent with the fact that the overall incidence rates of CRC in adults aged 50 and over declined by 32% between 2000 and 2013 [4,13]. One study of mortality in patients from the state of Wisconsin showed that Black/African American populations exhibited lower five-year survival rates for both colorectal and breast cancer diagnoses when compared to White and Hispanic populations [14].

While the evolution of CRC treatment is seeing overall increased survival rates, albeit gradual [15], there are still other factors at play that are interfering with these positive outcomes from occurring more frequently for Black patients. Several studies have identified potential factors, such as socioeconomic factors [5] and access to healthcare [11,12], that may explain why Black patients seem to have increased incidence and mortality rates. One analysis purported that the degree to which the mortality of cancer, in general, is higher in Black patients can be attributed to several things such as increased exposure to carcinogens such as cigarette smoking [16]. A study suggested that there may be an increased genetic predisposition at play to explain this, although this study was examining the occurrence of prostate cancer [17]. Black patients in the United States also tend to be diagnosed with cancer at later stages, which is likely due to lower screening rates and delays in seeking care for symptoms [18]. According to a previous study, an important observation was that Black patients presented more frequently with distant CRC than White patients [19]. Furthermore, Black patients are also less likely to receive appropriate cancer treatments and have more comorbid conditions [16]. However, it is important to note that these results are generalizations and may not apply to all Black and White patients.

Regarding this study’s observation of decreased mortality associated with Hispanic ethnicity, our data appear to be consistent with previous reports [20,21]. For instance, Miller et al. showed that compared to non-Hispanic Whites, Hispanics, male and female, have lower incidence rates of all cancers, but the gaps between the two ethnic groups have diminished over time [20]. The incidence rate ratio in colon cancer patients increased from 0.75 in 1995 to 0.91 in 2018, highlighting delayed declines among Hispanics partly due to slower uptake of screening [20]. Moreover, according to colorectal cancer statistics analyzed in 2020, death rates have declined slowly among Hispanics and American/Pacific Islanders when compared to non-Hispanic Black and non-Hispanic White patients, by about 1.8% per year from 2000/2001 to 2017 [20]. The death rate has remained stable since at least 1990 among AK Natives and AIs according to the same analysis [13].

Naturally, our study has some limitations. While the study aimed to provide analyses that controlled for several covariates, some residual confounding remains. For instance, it was not possible to control the analysis for insurance status, radiation therapy, or immune-modulating therapy. The data used rely on registry data, which is subject to coding errors and is not uniformly representative of the entire United States as not all states submit information to SEER. Additionally, the study did not account for differences in treatment received by Black and White patients, which may have contributed to the observed disparities in outcomes. This study works under the assumption that the standard of care was given to the patients who received treatment; most other studies have performed a degree of controlling for covariates, which is consistent with what this study has done. One potential covariate that other studies in the past have examined but does warrant a direct intervention to address is socioeconomic status. Some studies showed that socioeconomic status can negatively influence survival rates and incidence rates between races [5,7,11,14]. Also, there is some evidence that in situations in which Black patients in the most-affluent group had incidence rates of colorectal cancer similar to or exceeding those of White patients in the most-deprived group, Black patients were socially and materially worse off than White patients across different socioeconomic strata [5]. In one analysis, it was reported that when there is equal access to a healthcare system, there is no difference in overall survival between non-Hispanic Whites and non-Hispanic Blacks [22]. Therefore, it should stand to reason that instituting an equal access system to address health issues would result in an overall improvement.

## Conclusions

Our data found that Black patients had a significantly increased risk of developing and dying from malignant CRC compared with White patients. It is of great importance to understand why these trends exist to better guide public preventative measures and CRC awareness. Regardless of the explanations, there are several different avenues for interventions that can be taken to eliminate these disparities. Several proposals that can be made for this include increasing national awareness of the disease through targeted public health campaigns that highlight the risk factors and symptoms to be aware of within Black communities. Another recommendation is to increase access to screening and efforts for early detection, such as implementing policies and programs to ensure equitable access to these screenings, particularly in underserved communities; reducing the barriers to them, whether financial, insurance, or just the availability of it alone; and promoting the use of patient navigators or community health workers who can provide guidance, support, and follow-up for Black patients throughout the screening process. Policymakers should look and enact policies that lower socioeconomic disparities, such as promoting economic empowerment and financial stability in Black communities, and more overarching interventions, such as expanding coverage of Medicaid in states where it has not already been done, given that the lack of health insurance is a significant barrier to accessing appropriate care in a timely manner. Overall, addressing disparities in CRC outcomes is critical for improving public health and reducing healthcare disparities. The findings suggest that there may be underlying factors contributing to the increased risk of developing and dying from CRC in Black patients. This study identified race as an apparent increased risk factor for developing this condition, and from this, it must be understood whether there is an underlying genetic component or if this may be a result of external factors/variables that may predominantly affect Black populations.

Future research should focus on identifying the factors contributing to the disparities in CRC outcomes and developing interventions to reduce these disparities. Specifically, studies should investigate differences in genetics, diet, lifestyle, and access to healthcare that may contribute to the disparities in outcomes. Additionally, studies may investigate the role of differences in treatment received by Black and White patients and how these differences may contribute to disparities in outcomes. A more thorough analysis of the specifics of the treatment, such as specific combination chemotherapies or the use of specific surgical techniques, may reveal differences in outcomes or prolonged survival with certain regiments. One additional aspect of this study that can be attempted in future analyses is to group the data into more specific, shorter time periods. While the study aimed to examine the effects of the evolution of treatment on CRC survival rates, it used the incorporation of laparoscopic surgery into the guidelines for treatment as a point of separation between the two groups. Now, with advanced therapies such as immunomodulator therapy and more targeted chemotherapies, it would be interesting to see how the survival rates have been affected by the introduction of these more advanced therapies, as they are now becoming available in the armamentarium of anti-tumor treatments. 
